# Oral step-down for *Staphylococcus aureus* bacteraemia: An opportunity for antimicrobial stewardship?

**DOI:** 10.1016/j.clinpr.2022.100202

**Published:** 2022-11

**Authors:** Stephen Platts, Brendan A.I. Payne, D. Ashley Price, Lucia Pareja-Cebrian, Ulrich Schwab

**Affiliations:** a Medical School, Newcastle University, Newcastle-upon-Tyne, UK; b Translational and Clinical Research Institute, Newcastle University, Newcastle-upon-Tyne, UK; c Department of Infection and Tropical Medicine, The Newcastle-upon-Tyne Hospitals NHS Foundation Trust, Newcastle-upon-Tyne, UK; d Department of Microbiology and Virology, The Newcastle-upon-Tyne Hospitals NHS Foundation Trust, Newcastle-upon-Tyne, UK

**Keywords:** Staphylococcus aureus, Bacteraemia, Antimicrobial stewardship

## Abstract

**Objectives:**

Long courses of intravenous antimicrobial therapy are traditionally recommended for the treatment of methicillin sensitive *Staphylococcus aureus* bacteraemia (MS-SAB), but are not always completed in clinical practice. Early intravenous to oral antibiotic switch is a key component of antimicrobial stewardship. This study aimed to identify whether intravenous antibiotic duration may be safely reduced in MS-SAB.

**Methods:**

We performed a single-centre retrospective study of MS-SAB management. Successful outcome was defined as 90-day recurrence-free survival. Effect of intravenous antibiotic duration on 90-day recurrence risk was examined.

**Results:**

281 adult cases of MS-SAB were evaluated, of which 208 (74 %) had a successful outcome. 176 cases (63 %) received less than 14 days of intravenous antimicrobial therapy. Very short durations of intravenous therapy were associated with increased risk of recurrence (<7 days iv, 9.8 % recurrence; 7–13 days, 1.4 %; ≥14 days, 2.9 %; p 0.005). This effect was robust to sensitivity analysis for total antimicrobial therapy duration of 14 days. CRP reduction of at least 37 % from peak value at intravenous to oral antibiotic switch was associated with decreased risk of recurrence (<37 % CRP reduction, 12 % recurrence; >37 %, 2.0 %; p 0.001).

**Conclusions:**

Oral antimicrobial switch may allow safe reductions in duration of intravenous therapy in MS-SAB.

## Highlights

• Audit of nearly 300 cases of MSSA bacteraemia.
• 63 % received short-course intravenous therapy (<14 days).
• 7–13 days intravenous therapy showed equivalent recurrence risk to ≥14 days.
• Oral follow-on therapy is required until at least day 14.
• CRP reduction at oral step-down is predictive of recurrence risk.

## Introduction

Methicillin sensitive *Staphylococcus aureus* bacteraemia (MS-SAB) is associated with significant morbidity and mortality (Thwaites, 2010). Most guidance recommends treating MS-SAB with at least 14 days of intravenous antibiotics, but there is no high-quality evidence to guide practice (Holland et al., 2014, Tong et al., 2015). A recent report indicated ‘substantial practice variation’ in the management of MS-SAB (Liu et al., 2019). Attempts to classify patient populations who may be suitable for shorter courses of intravenous antibiotics (<14 days) have been proposed and are the subject of ongoing randomised controlled trials (RCT) (Kaasch et al., 2015).

Current antimicrobial stewardship guidelines advocate a continual ‘review and decide’ framework for inpatient administration of antibiotics. Every 48–72 h the antibiotics should be reviewed, including whether to switch route from intravenous to oral (Public Health England, 2015). Long courses of intravenous antibiotics are resource heavy and carry a higher risk of complications from intravenous catheters. However, the safety of intravenous to oral antibiotic switch in MS-SAB is not established. A Cochrane review concluded that there is some low quality evidence to suggest that the route of administration does not affect the rate of disease remission (Conterno and Turchi, 2013). More recently, the OVIVA trial demonstrated that oral antibiotic therapy was non-inferior to intravenous therapy in bone and joint infections without bacteraemia (Li et al., 2019). In stable patients with infective endocarditis, switching to oral antibiotics after a median of 17 days compared with 6 weeks of intravenous therapy was non-inferior (Iversen et al., 2018), and 22 % of these infections were with *S. aureus*. Other than defervescence and the absence of persistent bacteraemia, there are sparse data on the use of other biomarkers that might support an intravenous to oral switch in MS-SAB.

In our institution, intravenous antimicrobial therapy durations of less than 14 days are common in the treatment of MS-SAB. We therefore examined the safety of shorter courses of intravenous antimicrobial therapy for MS-SAB.

## Patients and methods

This was a retrospective single-centre observational study of episodes of MS-SAB in adult inpatients over a 2-year period, 2016–2017 inclusive. The total study population was all cases of MS-SAB aged ≥ 18 years at date of admission, excluding those cases deemed to be due to blood culture contaminants.

Favourable outcome was 90-day recurrence-free survival. Unfavourable outcomes were early (30-day) mortality, late (30–90 day) mortality, and recurrence by 90 days. For analyses of duration of antimicrobial therapy, only the outcome of 90-day recurrence risk was considered, as mortality or palliation may have truncated antimicrobial therapy duration. Based on the literature, we considered the expected rate of recurrence of MS-SAB to be about 5 %, with an upper limit of 10 %. We thus considered a duration of intravenous antimicrobial therapy to be potentially ‘safe’ if the one-sided 95 % upper binomial confidence limit for recurrence rate fell below 10 %. When considering duration of intravenous antimicrobial therapy, we performed sensitivity analysis for minimum total duration of 14 days of antimicrobial therapy.

Categorical factors were assessed by chi-squared or exact tests, as appropriate. Independent t tests were used to compare continuous variables. All statistical tests were 2-tailed and the threshold of statistical significance was p < 0.05. Those factors meeting marginal significance (p < 0.1) on univariate analysis were included in a multivariate binary logistic regression model. In order to determine a putative cut-off value for CRP reduction at oral antibiotic switch, ROC curve analysis was used with Kolmogorov-Smirnov (K-S) statistic.

The study was registered with our institutional clinical governance department and was deemed to be exempt from the requirement for ethics committee approval or patient-level consent.

## Results

### Patient demographics

After exclusion of four blood culture isolates deemed to be contaminants, the total study population comprised 281 adult cases of MS-SAB. Mean age was 60.0 years (SD 19.1) and 183 (65 %) were male. 144 (51 %) of infections were classified as healthcare-associated (HAI, blood culture drawn at >48 h post-admission). The most common attributed source of infection was intravascular catheters (IVC, 70 cases, 25 %). 40 cases (14 %) were associated with bone or joint infection (BJI), 17 (6 %) with infective endocarditis, and 16 (6 %) with dialysis site infection. 16 cases (6 %) were in intravenous drug users.

### Outcomes of MS-SAB

In total, 208 cases (74 %) were classed as having favourable outcome (90-day recurrence-free survival). 14 cases (5 %) had recurrence of MS-SAB within 90 days, 43 (15 %) had early mortality (within 30 days), and 16 (6 %) had late mortality (between 30 and 90 days). On unadjusted analysis, favourable outcome was associated with younger age (mean age (SD), 57.0 years (SD 18.5) with favourable outcome, 68.7 years (18.2) with unfavourable, t 4.67, p < 0.001), community acquired infection (CAI 83 %, HAI 65 %, χ^2^(1) 11.21, p 0.001), and BJI (BJI 93 %, other site of infection 82 %, Fisher’s Exact p 0.003). Recurrence of MS-SAB was associated with dialysis site infection only (dialysis site infection 19 % recurrence, other site of infection 4 %, Fisher’s Exact p 0.038, Table 1). Early mortality was associated with age (deceased 71.4 years (16.0), alive 58.0 (18.9), t 4.38, p < 0.001), HAI (HAI 21 % mortality, CAI 9 %, Fisher’s Exact p 0.004) and infections other than BJI (BJI 2.5 %, other source 17 %, Fisher’s Exact p 0.015). 238 cases were alive at 30 days and included in analysis of late mortality. Late mortality was associated with increased age only (deceased 73.1 years (15.2), alive 56.9 (18.7), t 3.39, p 0.001).

**Table 1:** Predictors of recurrent MS-SAB at 90 days.

Factor	Recurrent MS-SAB	p value (adj.)
Gender (M, F)	7/183 (3.8 %), 7/98 (7.1 %)	ns
Onset (HAI, CAI)	9/144 (6.3 %), 5/137 (3.6 %)	ns
IVDU	1/16 (6.3 %)	ns
*Source*		
IVC	2/70 (2.9 %)	ns
Dialysis site	3/16 (19 %)	**0.038 (0.020)**
Surgical site	3/28 (11 %)	ns
Infective endocarditis	2/17 (12 %)	ns
Bone/joint infection	1/40 (2.5 %)	
Undetermined	2/35 (5.7 %)	ns
*Antimicrobial therapy*		
iv duration (d) <7, 7–13, ≥14	10/102 (9.8 %), 1/74 (1.4 %), 3/105 (2.9 %)	**0.005 (0.013)**
Total duration (d) <14, ≥14	6/71 (8.5 %), 8/210 (3.8 %)	0.12
CRP ratio (low, high)	4/197 (2.0 %), 9/73 (12 %)	**0.001 (0.034)**

### Antimicrobial therapy

176 cases (63 %) received less than 14 days of intravenous antimicrobial therapy, and 102 (36 %) received less than 7 days. 186 cases (66 %) received less than 28 days of total antimicrobial therapy (intravenous and/or oral), and 71 (25 %) received less than 14 days. 232 cases (83 %) received at least one dose of an intravenous beta-lactam antimicrobial, which was intravenous flucloxacillin in 176 cases (63 %). As expected, there was variation in length of antimicrobial therapy course with indication. However, the only factors significantly associated with intravenous antibiotic duration of at least 7 days were BJI (BJI 85 % ≥7 days intravenous, other source 60 %, χ^2^(1) 9.15, p 0.002) and male gender (male 68 % ≥7 days, female 55 %, χ^2^(1) 4.81, p 0.028). 172 cases had intravenous to oral antimicrobial switch. Agents used for oral follow-on therapy are shown in Fig. 2.

**Fig. 1:**
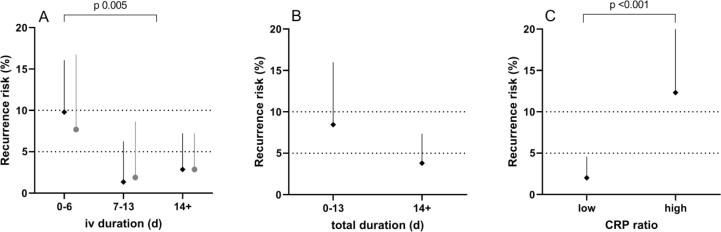
**Intravenous to oral antimicrobial therapy switch and probability of recurrence. A.** Very short duration intravenous antibiotic therapy (<7 days) was associated with increased risk of recurrent MS-SAB within 90 days. Durations of 7–13 days showed no increased risk compared with ≥14 days. **B.** Total duration of antimicrobial therapy (intravenous plus oral) of less than 14 days was associated with upper confidence limit exceeding 10 %. **C.** Low CRP ratio (reduction of at least 37 % from peak value) was associated with reduced risk of recurrence. Dark dots show whole dataset. Pale dots show sensitivity analyses. Error bars show one-sided 95 % binomial CI. Dotted lines show expected range of MS-SAB recurrence rate (5–10 %).

**Fig. 2:**
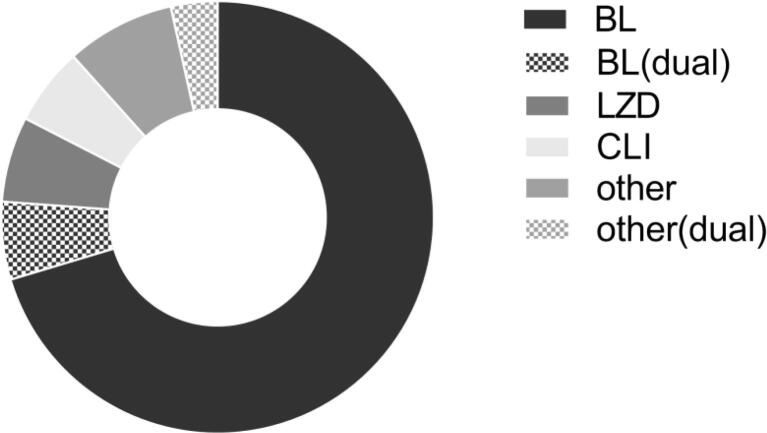
**Oral antimicrobial follow-on agents in MS-SAB.** Follow-on oral anti-staphylococcal therapy after initial intravenous therapy (n = 172). Where more than one oral anti-staphylococcal agent used, the agent used immediately after intravenous to oral switch is shown. BL, beta-lactam; BL(dual), dual oral antimicrobial therapy including a BL; CLI, clindamycin; LZD, linezolid; other(dual), dual oral antimicrobial therapy not including a BL, CLI or LZD.

Risk of recurrent MS-SAB was strongly associated with duration of intravenous antimicrobial therapy (<7 days iv, 9.8 % recurrence; 7–13 days, 1.4 %; ≥14 days, 2.9 %; χ^2^(1) 7.86, p 0.005 for comparison of < or ≥7 days, Fig. 1**A**). The same effect was seen, but slightly attenuated, in a sensitivity analysis including only those 202 cases receiving at least 14 days of total antimicrobial therapy (<7 days iv, 7.7 % recurrence; 7–13 days, 1.9 %; ≥14 days, 2.9 %; χ^2^(1) 2.84, p 0.09). Amongst those cases receiving less than 7 days of intravenous therapy, recurrence was particularly high with intravenous duration of less than 2 days (19 %) compared with 2–6 days (4.5 %).

Total duration of antimicrobial therapy is likely to be largely a function of underlying source of infection. However, where short courses of intravenous antimicrobial therapy are used, a minimum total duration of antibiotic treatment may be required for associated MS-SAB. The effect of total antimicrobial therapy duration on risk of recurrence of MS-SAB did not reach statistical significance (<14 days total duration, 8.5 % recurrence; ≥14 days, 3.8 %; Fisher’s Exact p 0.13, Fig. 1**B**). However, for a total antimicrobial therapy duration of less than 14 days, the upper confidence limit of recurrence rate was 16 %. 158 cases of MS-SAB were treated with at least 14 days of antimicrobial therapy of which at least 7 days was intravenous. Recurrence rate in this group was very low (2.5 %, upper confidence limit 5.7 %).

On multivariate analysis, both intravenous antimicrobial treatment duration of <7 days (p 0.013) and dialysis site infection (p 0.020) were independently associated with increased risk of recurrence of MS-SAB, in a model that also included BJI and gender.

### Choice of intravenous antimicrobial therapy

Amongst the 245 cases receiving at least 2 days of any intravenous antimicrobial therapy, recurrence risk did not differ significantly according to whether an intravenous beta-lactam (ivBL) was used (ivBL, 2.7 % recurrence; no ivBL, 4.3 %; Fisher’s Exact p 0.50).

### C-reactive protein

In clinical practice, a declining CRP is commonly used as a biomarker of improvement in the context of infection. The highest CRP and the CRP closest to intravenous to oral antibiotic switch were used to calculate a ‘CRP ratio’ at oral switch (data available for 270 cases). On ROC analysis, CRP ratio was moderately predictive of recurrence risk (area under ROC curve, 0.72). The optimal CRP ratio cut-off was calculated as 0.63 (i.e. a reduction in CRP at intravenous to oral antibiotic switch of at least 37 % from peak). Thus, risk of recurrence was 12 % where CRP had decreased by less than 37 % at oral antibiotic switch compared with 2.0 % where CRP reduction was greater than 37 % (Fisher’s Exact p 0.001). This effect remained independently significant (p 0.034) after adjusting for intravenous antibiotic duration (greater than 7 days), dialysis site infection, BJI and gender.

## Discussion

In our centre, the majority of cases of MS-SAB received less than 14 days of intravenous antimicrobial therapy. We found that intravenous antibiotic durations of 7–13 days showed equivalent recurrence risk to the ‘standard’ intravenous duration of 14 days or more. Intravenous durations of less than 7 days fell outside our pre-specified acceptable limit, as we could not exclude that recurrence rate exceeded 10 %, although most of this effect was accounted for by extremely short intravenous courses of less than 2 days. Total antimicrobial therapy duration is likely to be predominantly influenced by underlying source of infection, and may not be directly relevant to the successful management of the associated bacteraemia. However, total antibiotic treatment durations of less than 14 days had an upper limit for recurrence rate which fell outside the acceptable range. Based on our data, we therefore suggest that where a shorter intravenous antibiotic duration of 7–13 days is used for MS-SAB, follow-on oral antibiotics should be used to achieve a total antibiotic duration of at least 14 days.

Our study therefore gives support for the role of oral antibiotic switch in MS-SAB, something for which there is a paucity of published literature. Smaller studies have examined oral step-down after a median of 5 or 6 days intravenous therapy in low-risk SAB in adults and children, with low rate of relapse (Bupha-Intr et al., 2020, Gunter et al., 2022). Our larger study suggests that oral step-down may also be applicable to a wider group of MS-SAB patients.

CRP is very commonly used as a biomarker of treatment response to antimicrobial therapy, but there is a lack of data for its application to MS-SAB. Our data suggest that a substantial reduction in CRP from peak value may be a reasonable indicator of successful response to therapy and may help facilitate a safe early oral switch.

The retrospective and observational nature of our study inevitably leads to some limitations, and caution should be used in attributing causality. Antimicrobial therapy choices are individualised, especially in complex infections and where risk of complications is deemed higher. These findings must therefore be replicated in RCT. 13 % of laboratory-documented episodes of MS-SAB were excluded due to insufficient data. In addition, we cannot exclude the possibility that we have underestimated the rate of recurrence as some patients may have presented to another hospital. In this study we elected to include all adult cases of MS-SAB, rather than focus solely on ‘uncomplicated’ cases, as is the more common approach in the published literature. There were several reasons behind this decision. Cases were very rarely explicitly classified as ‘uncomplicated’ in clinical practice, and half of cases could not be classified as complicated or uncomplicated even with retrospect. In those cases that could be classified, this appeared to have no effect on the duration of antibiotic therapy given. It is recognised that ‘uncomplicated’ SAB is essentially a diagnosis of exclusion, and complications (such as metastatic infection) are frequently not recognised at the time of initial assessment, and therefore may not be of much value in guiding the initial management plan.

These data suggest that antimicrobial stewardship can be safely applied to MS-SAB. Our findings support the case for further RCT to confirm the safety of early intravenous to oral antibiotic switch in MS-SAB. Given the complex factors dictating antibiotic decision making in MS-SAB, tools could be developed within electronic patient records to guide antimicrobial stewardship decisions in MS-SAB.

### Inserts

HAI, healthcare-associated infection; CAI, community-acquired infection; IVDU, intravenous drug user; IVC, intravascular catheter; CRP ratio, greater or less than 37 % fall from peak value at oral switch. Adjusted model: gender, bone/joint infection, dialysis site infection, iv duration ≥7 days.

## Declaration of Competing Interest

The authors declare that they have no known competing financial interests or personal relationships that could have appeared to influence the work reported in this paper.
